# Supplementary figures and images for: Assessment of Gene Set Enrichment Analysis using curated RNA-seq-based benchmarks
Source: PLoS One. 2024 May 16;19(5):e0302696. doi: 10.1371/journal.pone.0302696 (PMC11098418; doi:10.1371/journal.pone.0302696)

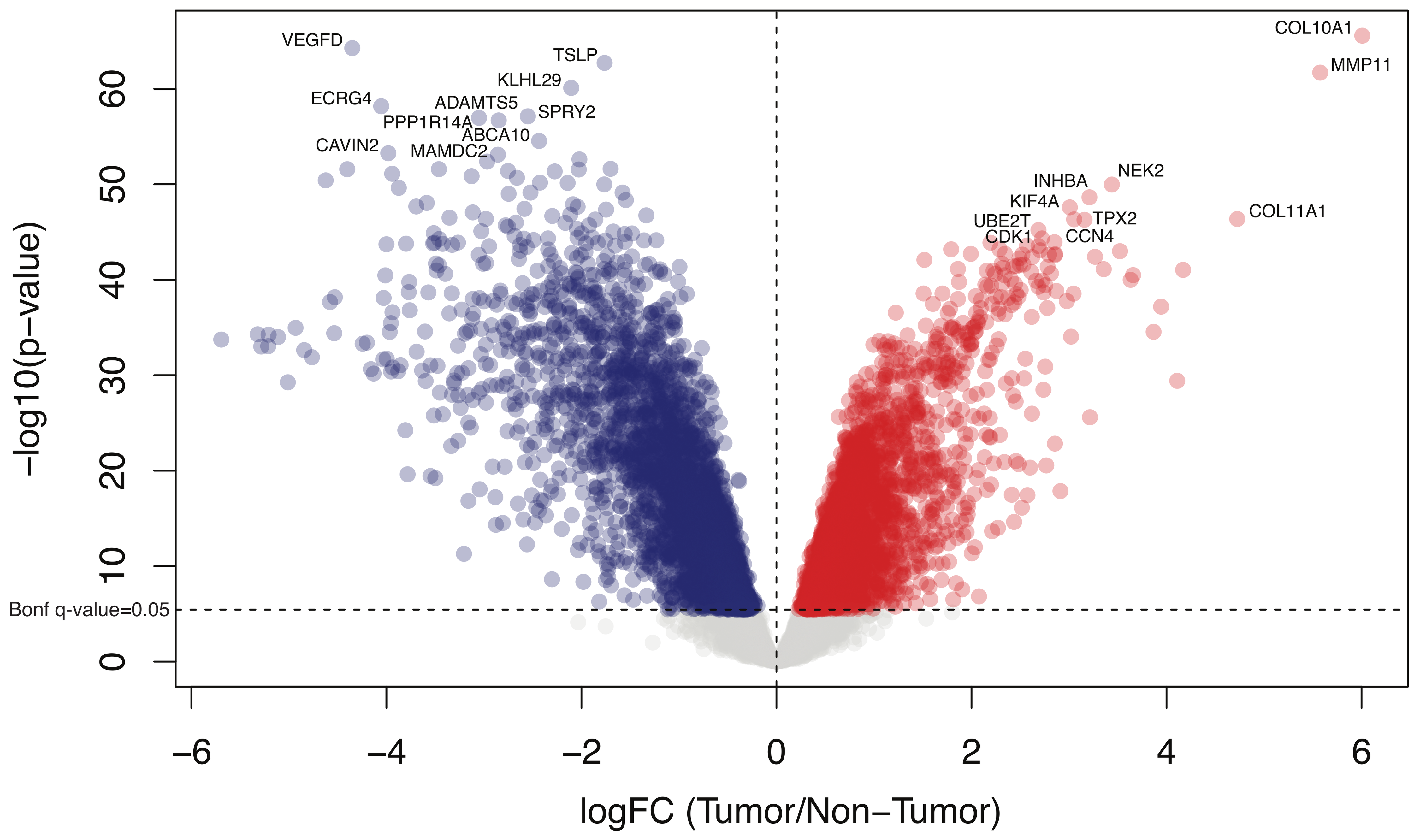

Supplement: S1 Fig — Volcano plot showing significant genes over-expressed in primary-tumor (red) or non-tumor (blue) tissue based on Bonferroni-adjusted q—value < 0.05. Labels for the top ten genes on either side are also shown. (TIF) [file pone.0302696.s007.tif]

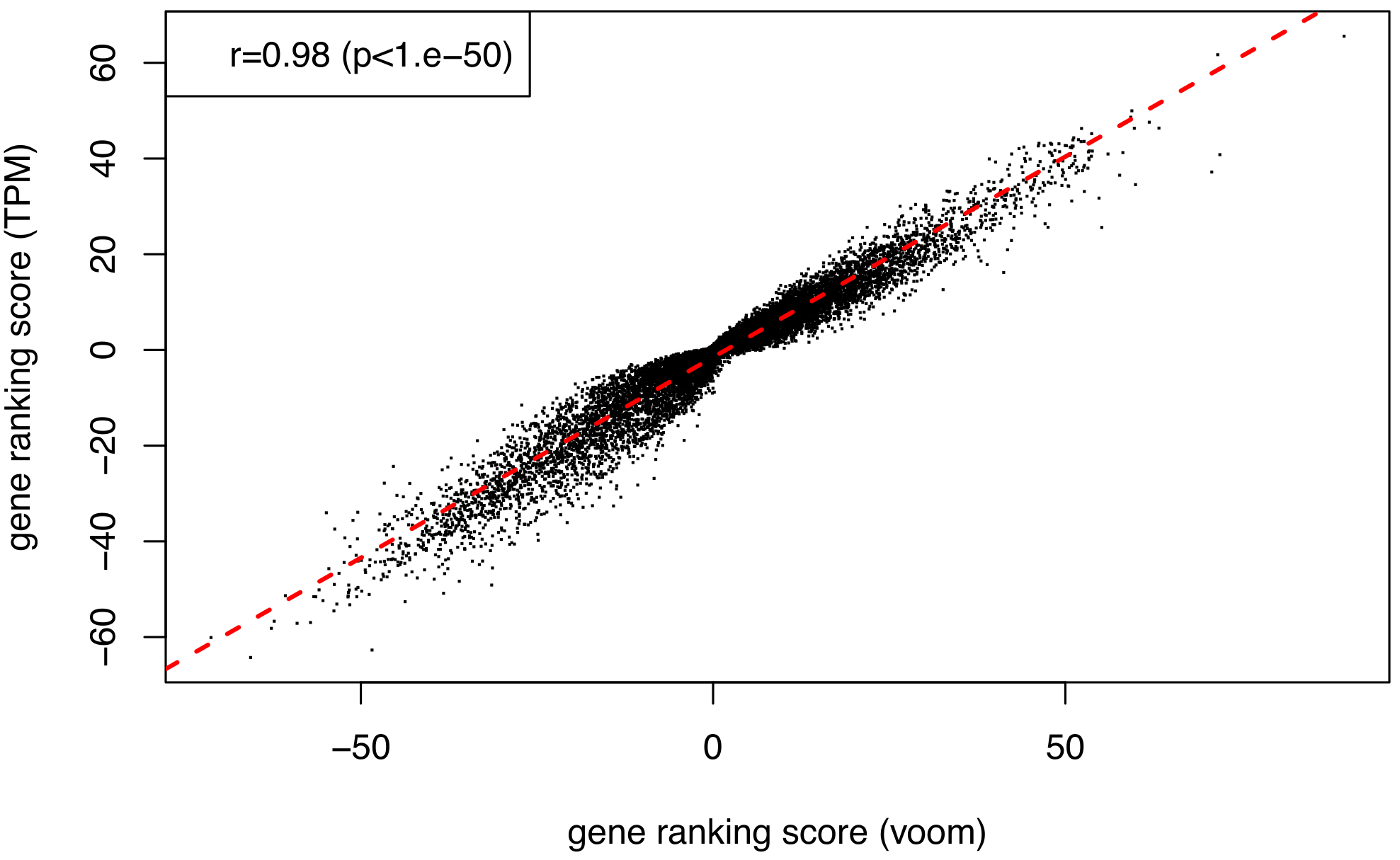

Supplement: S2 Fig — DGE was assessed from 228 paired primary-tumor vs non-tumor breast cancer samples from TCGA-BRCA. The linear fit is shown by a dashed red line. Spearman’s correlation estimate and p-value are shown in the legend. (TIF) [file pone.0302696.s008.tif]

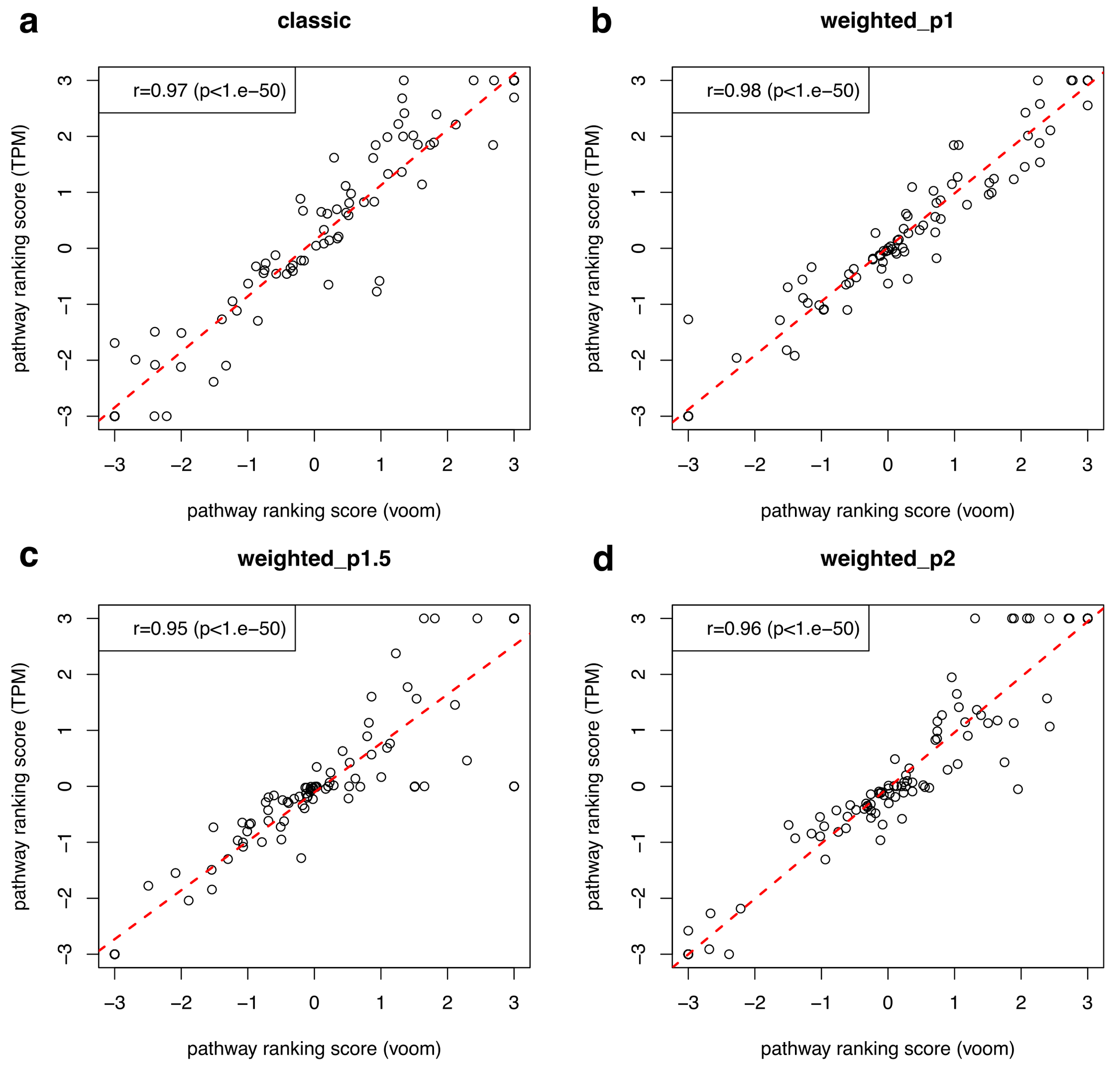

Supplement: S3 Fig — DGE was assessed from 228 paired primary-tumor vs non-tumor breast cancer samples from TCGA-BRCA. Target pathways were analyzed using GSEA with different enrichment statistics: (a) classic (unweighted); (b) weight parameter p = 1; (c) weight parameter p = 1.5; (d) weight parameter p = 2. Pathway ranking scores were defined as −log_10(p—value)*sign(ES), where p-values were empirically determined via gene-set permutations and ES represented gene set enrichment scores. Because we used 1000 permutations for GSEA’s null model, we adopted p—value = 0.001 as lower threshold, which implies that pathway ranking scores are constrained to the [−3, 3] range. Linear fits are shown by dashed red lines. Spearman’s correlation estimates and p-values are shown in the legends. (TIF) [file pone.0302696.s009.tif]

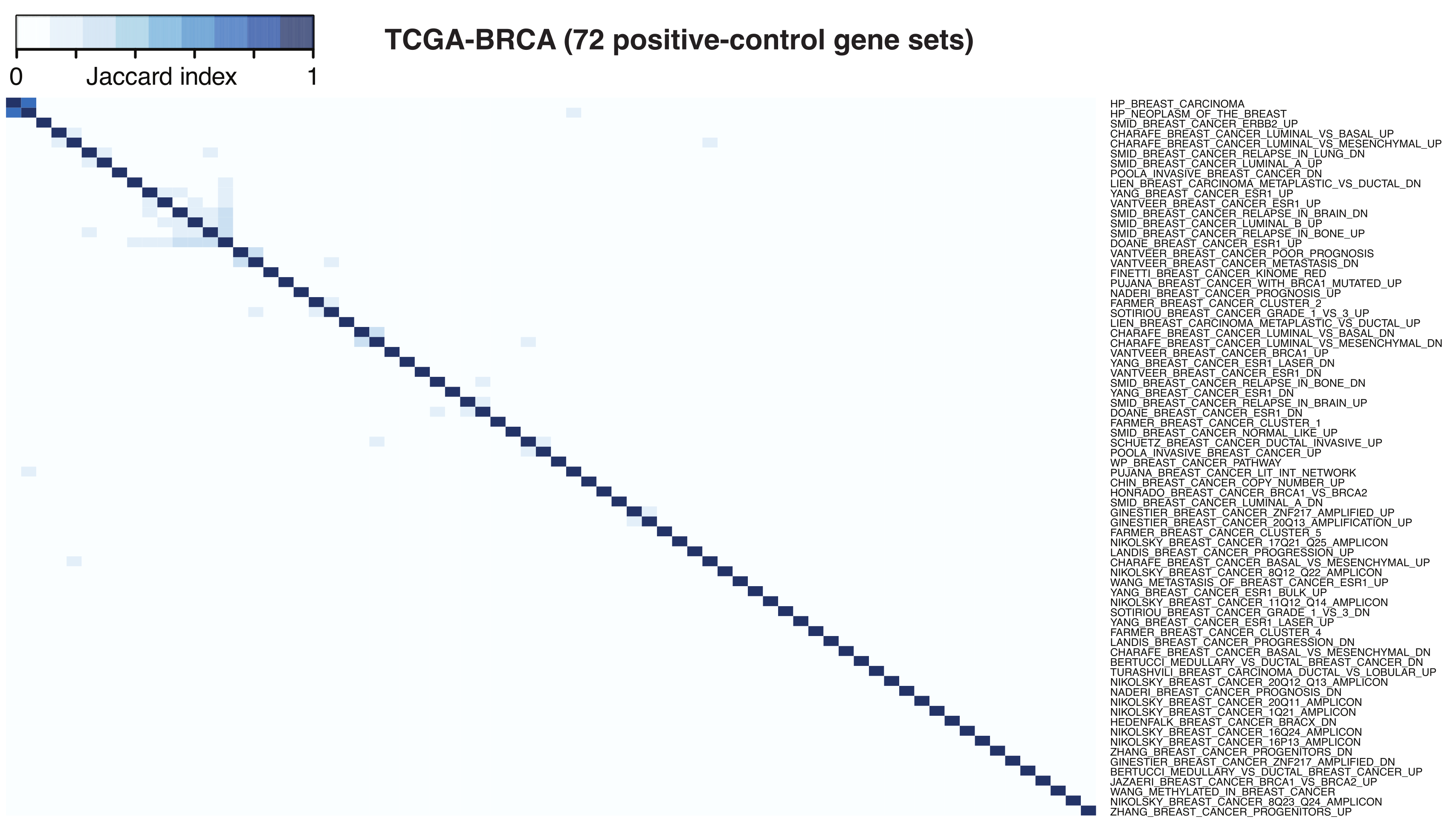

Supplement: S4 Fig — (TIF) [file pone.0302696.s010.tif]

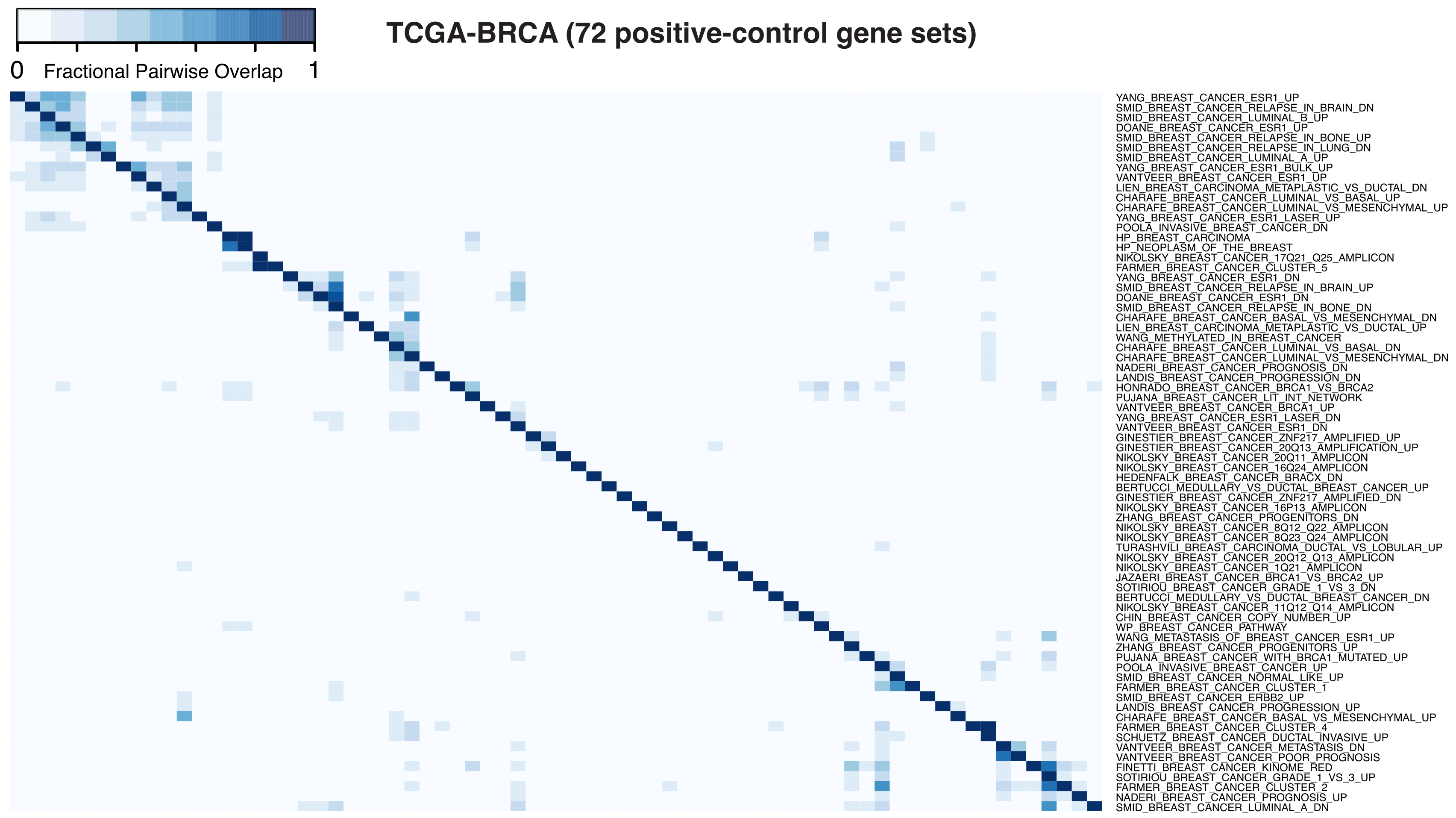

Supplement: S5 Fig — (TIF) [file pone.0302696.s011.tif]

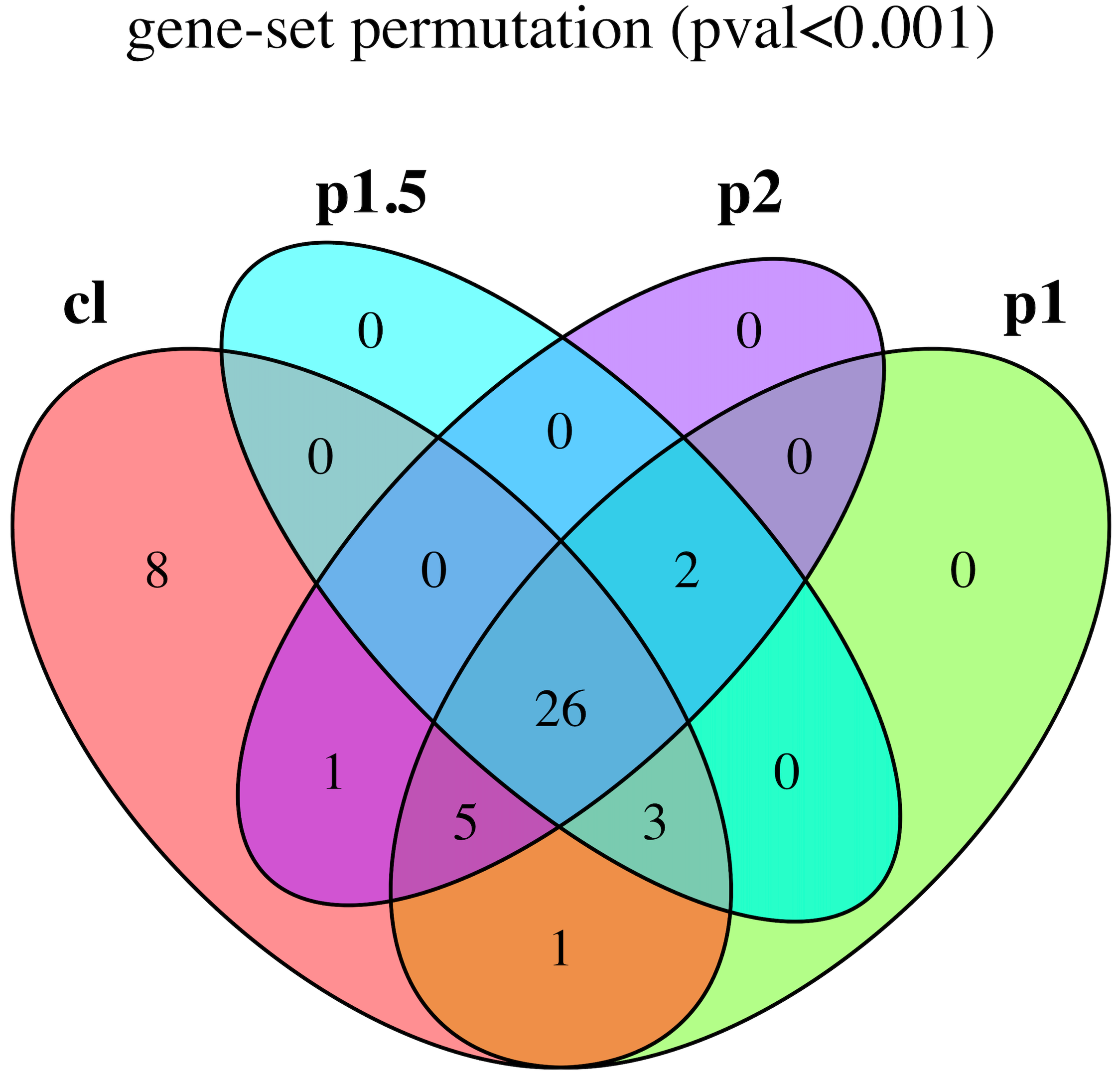

Supplement: S6 Fig — Gene-set permutation with p—value < 0.001. (TIF) [file pone.0302696.s012.tif]

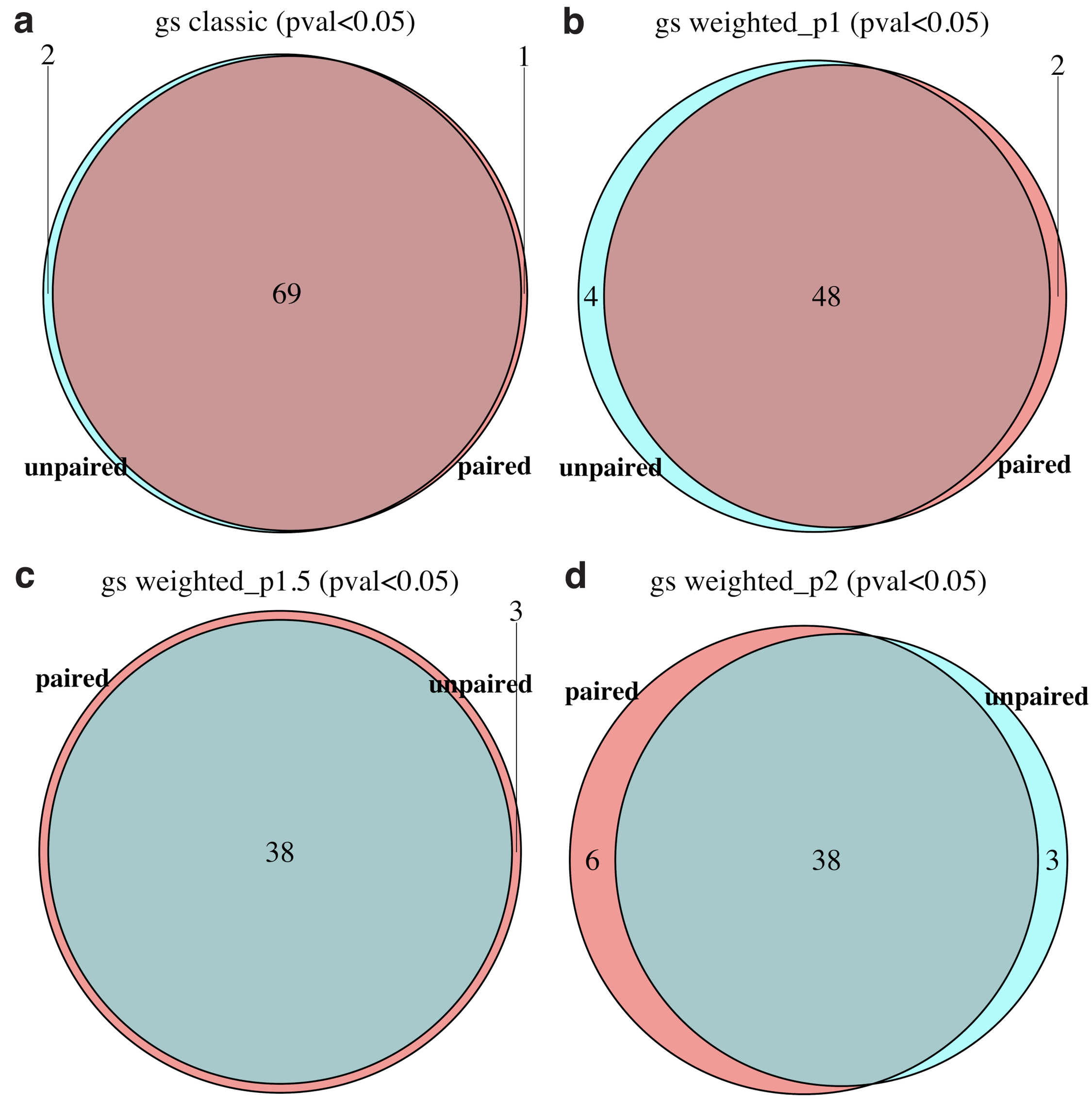

Supplement: S7 Fig — The significance criterion was p—value < 0.05. (a) Classic (unweighted). (b) Weight parameter p = 1. (c) Weight parameter p = 1.5. (d) Weight parameter p = 2. (TIF) [file pone.0302696.s013.tif]

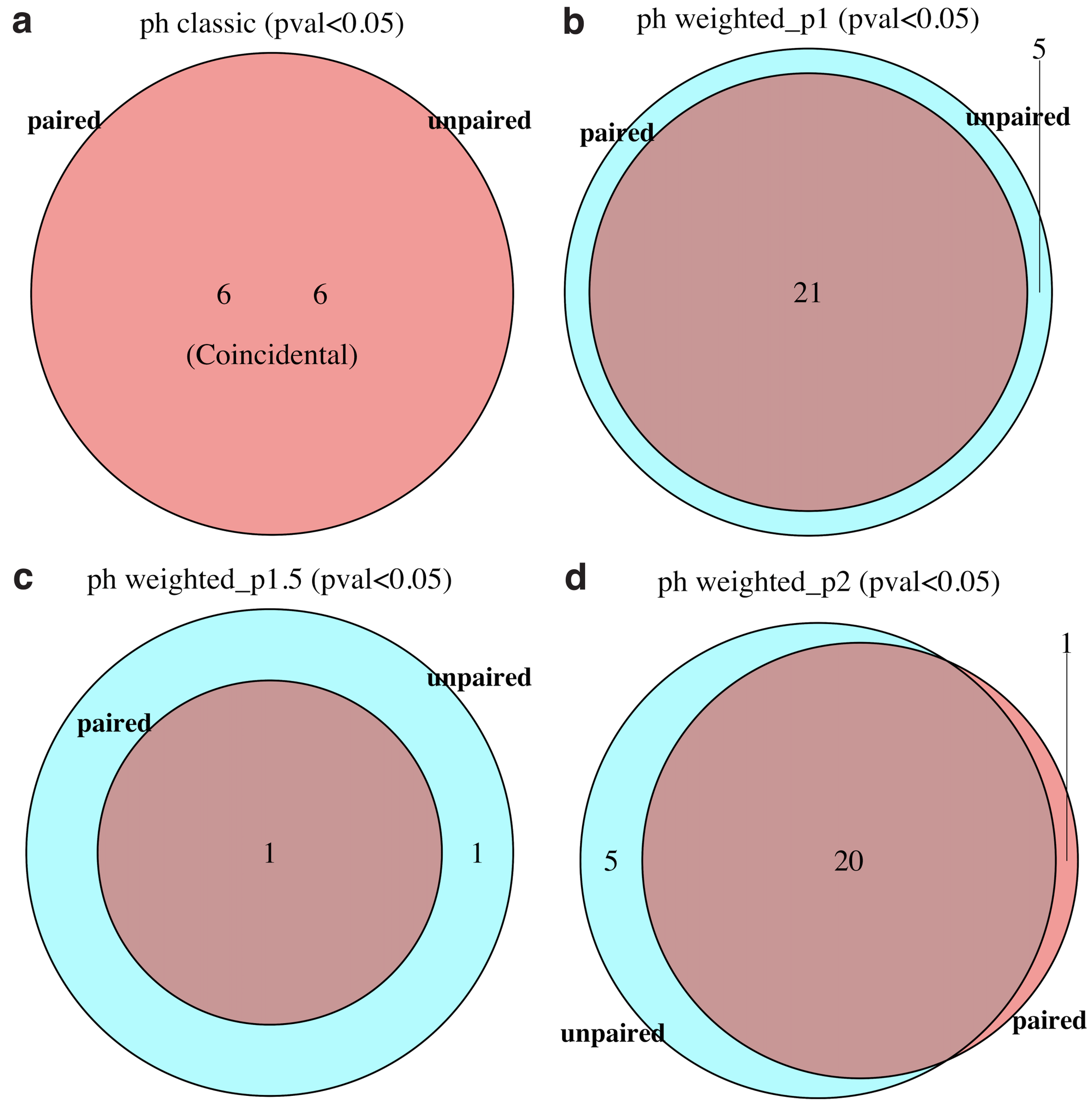

Supplement: S8 Fig — The significance criterion was p—value < 0.05. (a) Classic (unweighted). (b) Weight parameter p = 1. (c) Weight parameter p = 1.5. (d) Weight parameter p = 2. (TIF) [file pone.0302696.s014.tif]

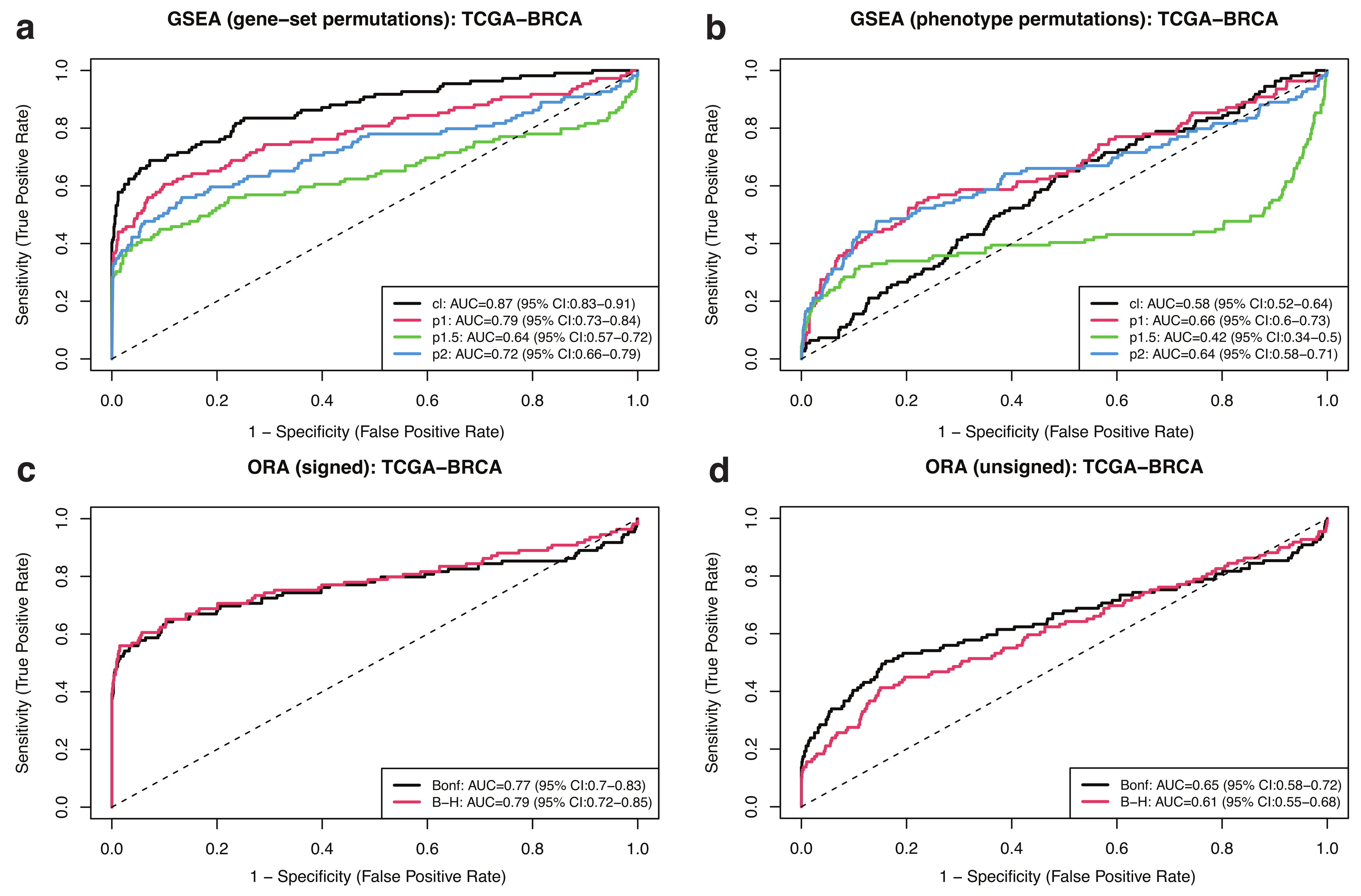

Supplement: S9 Fig — (a) Gene-set permutation GSEA. (b) Phenotype permutation GSEA. (c) Signed ORA. (d) Unsigned ORA. GSEA approaches used different enrichment statistics, as indicated. ORA approaches used Bonferroni and Benjamini-Hochberg (B-H) adjusted p-values as different inclusion criteria to select differentially expressed genes, as indicated. (TIF) [file pone.0302696.s015.tif]

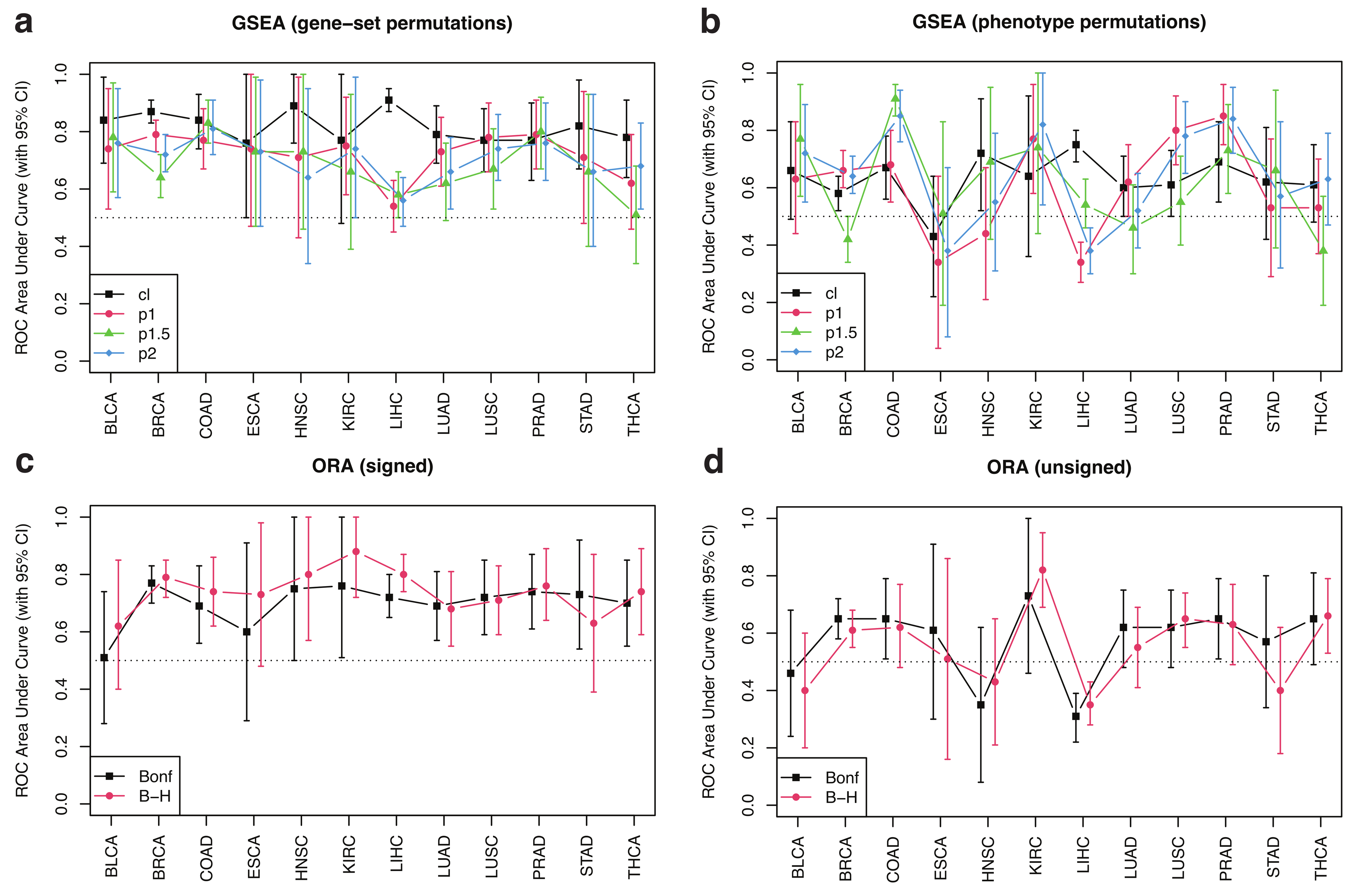

Supplement: S10 Fig — (a) Gene-set permutation GSEA. (b) Phenotype permutation GSEA. (c) Signed ORA. (d) Unsigned ORA. GSEA approaches used different enrichment statistics, as indicated. ORA approaches used Bonferroni and Benjamini-Hochberg (B-H) adjusted p-values as different inclusion criteria to select differentially expressed genes, as indicated. (TIF) [file pone.0302696.s016.tif]

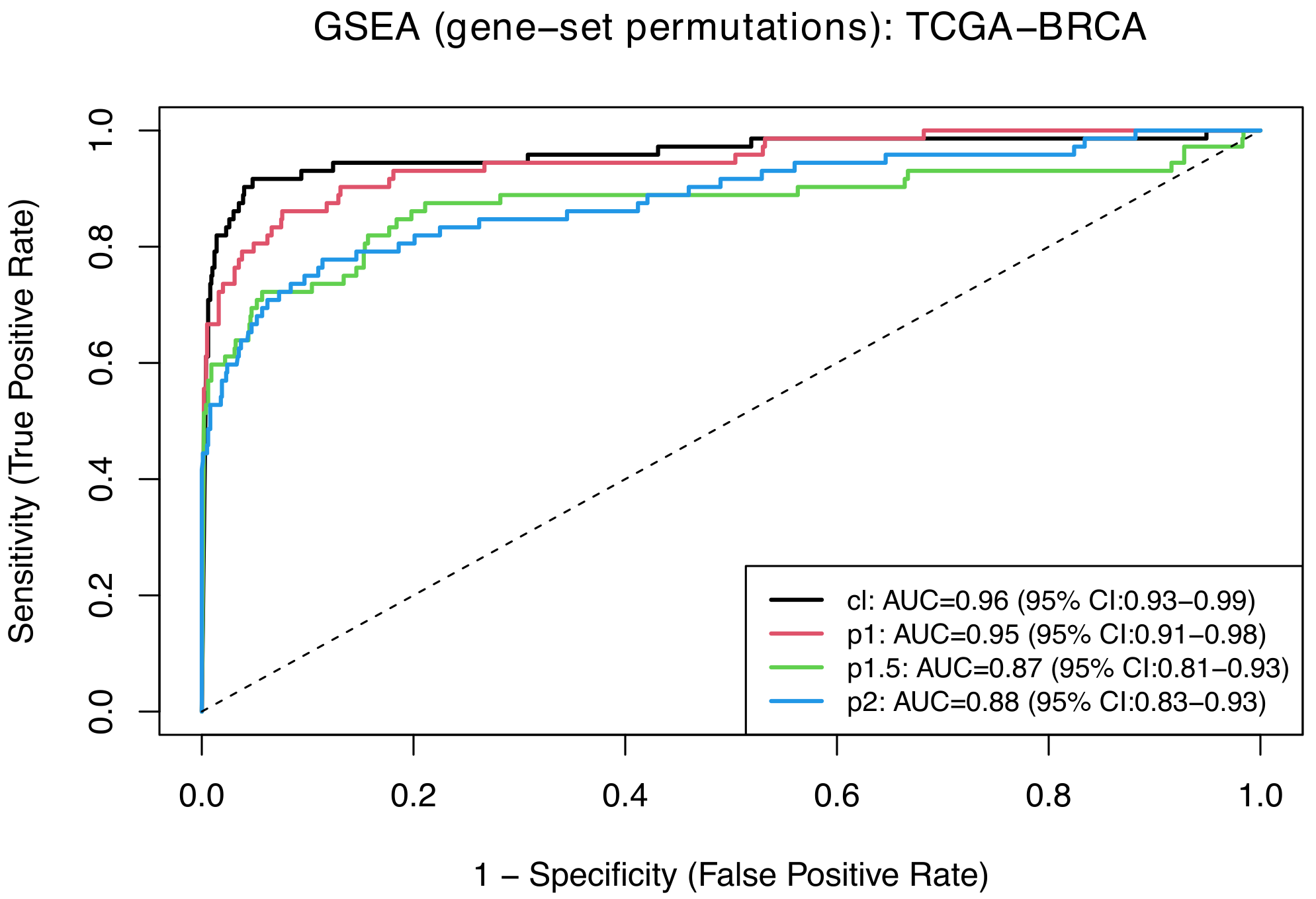

Supplement: S11 Fig — Results obtained by an alternative gene expression analysis derived via the edgeR-voom-limma pipeline described in Sect. 2.2. (TIF) [file pone.0302696.s017.tif]

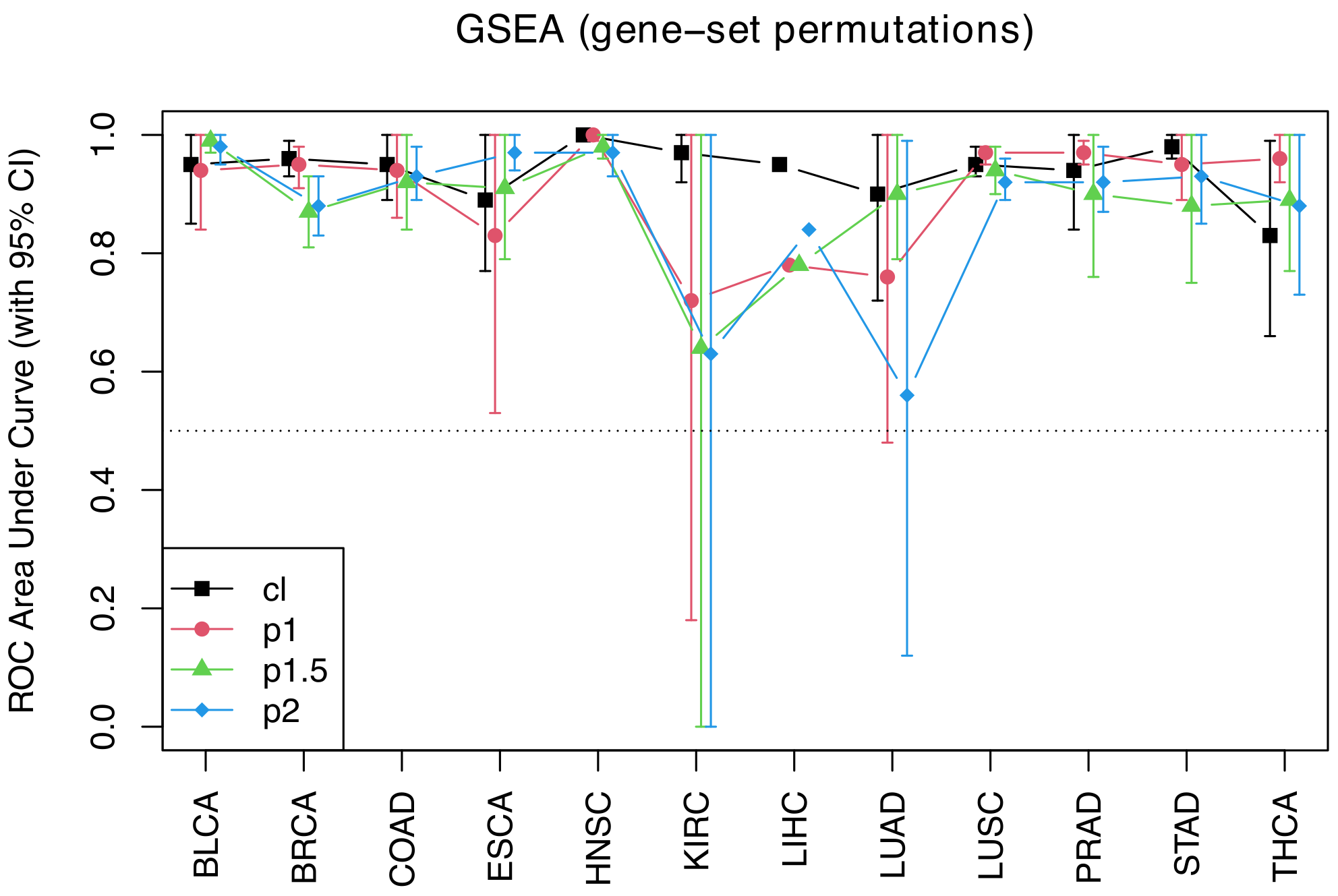

Supplement: S12 Fig — Results obtained by an alternative gene expression analysis derived via the edgeR-voom-limma pipeline described in Sect. 2.2. (TIF) [file pone.0302696.s018.tif]
